# Ti_3_C_2_T_x_ (MXene)‐Polystyrene Based Passivation for Perovskite Solar Cells With Enhanced Stability

**DOI:** 10.1002/smll.202513734

**Published:** 2026-05-24

**Authors:** Selengesuren Suragtkhuu, Purevlkham Myagmarsereejid, Isaac Etchells, Paul E. Shaw, Yu Lin Zhong, Munkhbayar Batmunkh

**Affiliations:** ^1^ School of Environment and Science Griffith University Nathan Queensland Australia; ^2^ Centre for Organic Photonics & Electronics School of Chemistry & Molecular Bioscience The University of Queensland Brisbane Queensland Australia

**Keywords:** MXene, perovskite solar cells, photovoltaic, polystyrene, surface passivation

## Abstract

Perovskite solar cells are the fastest advancing photovoltaic technology, but their long‐term durability remains a major barrier to commercialization. Surface passivation is a promising and straightforward approach to improve the stability of PSCs while enhancing their performance through surface defect modifications. In this work, we develop a novel passivation layer by employing conductive few‐layer two‐dimensional (2D) Ti_3_C_2_T_x_ (MXene) sheets and hydrophobic polystyrene (PS). This passivation layer not only enables improved charge transfer through nanoscale conductive domains, but also displays excellent moisture tolerance for PSCs. As a result, this newly designed passivation enhanced the crystallinity, reduced the surface defects, and improved the charge transports in PSCs. The PSC device fabricated with PS and MXene (PSMX) passivation achieved a power conversion efficiency (PCE) of 20.01%, outperforming the cells with PS‐only passivated perovskite films. Remarkably, unencapsulated PSMX passivated devices preserved more than 93% of their initial PCEs after aging for 30 days in ambient conditions (40%–50% relative humidity), demonstrating excellent operational stability. These results showcase the effectiveness of integrating conductive 2D materials into hydrophobic polymers for surface passivation, offering a practical pathway toward high‐performance and stable PSCs.

## Graphical abstract

A new class of surface passivation strategy by integrating conductive 2D Ti_3_C_2_T_x_ and hydrophobic polystyrene for stable perovskite solar cells is introduced in this work. Polystyrene‐MXene passivated devices retained more than 93% of their initial efficiencies after storing in ambient air with high humidity conditions for 30 days.

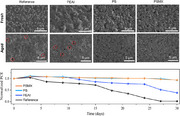

## 1 Introduction

Organometallic halide perovskite solar cells (PSCs) have received tremendous attention from both academic and industrial communities. Within two decades, the power conversion efficiency (PCE) of PSCs has soared from 3.8% to >27% [1], making them the fastest advancing photovoltaic (PV) technology in history. However, PSCs suffer from long‐term operational stabilities under ambient conditions, limiting their potential opportunities for widespread commercialization. It is well documented that perovskite materials deteriorate rapidly in humid environments or other harsh conditions [2, 3, 4]. To address this issue, several strategies have been developed, including compositional engineering [5], 2D perovskite [6, 7], interface engineering [8, 9], surface passivation [10, 11, 12], and encapsulation [13, 14].

Surface passivation is widely used as an effective and straightforward method to improve surface defects in perovskite films, thereby enhancing device performance and stability [15, 16, 17, 18]. In particular, large organic halide salts such as n‐butylammonium iodide (n‐BAI) [19, 20, 21] and phenylethylammonium iodide (PEAI) [10, 22, 23] have attracted significant research interest and have often been used for highly efficient PSCs. Moreover, integrating long‐chain ammonium halides to form 3D/2D perovskite heterostructures has emerged as a highly effective strategy to mitigate ion migration, iodine vacancies, surface defects, and non‐radiative recombination, and thus enhancing the operational stability and PV efficiency of PSCs [24, 25, 26, 27]. However, the key challenges for these traditional 3D/2D perovskites include the poor conductivity and deprotonation of the 2D layers [28, 29].

Alternatively, low‐cost insulating polymers on the surface of the perovskite film have been proven to effectively passivate the perovskites, which resulted in not only increased efficiency, but also improved stability [30, 31, 32]. Recently, Martinez et al. [33], introduced a very thin layer of poly(methyl methacrylate (PMMA) as a surface passivation on the perovskite film to suppress the surface defects, achieving the efficiency and stability enhancement. Wang et al. [16], explored the effect of three different polymers, including polystyrene (PS), polytetrafluoroethylene (PTFE), and polyvinylidene‐trifluoroethylene copolymer (PVDF‐TrFE), as a passivation layer for perovskite films. These passivation strategies displayed excellent enhancements in the PV efficiencies and stabilities of PSCs. These studies demonstrate the positive impact of a polymer passivation layer for perovskite films, leading to the fabrication of PSCs with improved stability as they serve as an encapsulation layer to prevent the perovskite films while also passivating the surface defects. However, due to the insulating nature of the polymer layer, the interfacial carrier transports in perovskite films are often weakened, increasing the device series resistance and thus resulting in limited fill factor (FF) values in the fabricated devices [34, 35, 36]. Therefore, research into designing a conductive polymer passivation layer for perovskite films is expected to improve not only stability but also the efficiency of PSCs.

Two‐dimensional (2D) materials with their unique properties such as graphene, transition‐metal dichalcogenides (TMDs), phosphorene, and antimonene have attracted a great deal of attention for PSCs [37, 38, 39]. Very recently, Lou et al. [40], designed an efficient passivation layer of polymer and 2D material composite using PMMA and fluorinated graphene (PMMA: graphene), which enabled the preparation of high efficiency PSCs with a PCE of 22.91%. This device with PMMA: graphene showed excellent stability, maintaining over 90% of the initial PCE after 1200 h of aging at a relative humidity (RH) of 35% at room temperature. Subsequently, a hybrid 2D reduced graphene oxide (rGO) nanosheets and MoS_2_ quantum dots was used as an interfacial layer between the perovskite and the hole transporting layer (HTL), yielding efficiencies above 20% with excellent stability, as compared to 17% efficiency for the reference device [41]. Moreover, Bati and colleagues employed a nickel (Ni) atom‐doped 2D phosphorene as a surface passivation layer. As a result, treated devices achieved significantly improved PCEs (>22%) with negligible hysteresis as compared to untreated PSCs [42]. As a novel class of 2D materials, MXene has emerged as a promising candidate for various applications [43, 44, 45, 46, 47] due to its excellent properties such as highly tunable electronic properties [48], hydrophilic surface [49], and high conductivity [50]. Owing these fascinating properties, it is reasonable to expect a highly efficient passivation layer by integrating conductive Ti_3_C_2_T_x_ (MXene) and polymer materials for PSC.

Herein, we synthesized conductive Ti_3_C_2_T_x_ MXene nanosheets and incorporated them with hydrophobic PS polymer to form an efficient surface passivation layer for PSCs. The optimized passivation layer enabled improved PV efficiencies while showing outstanding device stabilities, retaining more than 93% of the initial PCEs after being stored in ambient air (40%–50% RH) for 30 days. A comprehensive set of measurements was performed to evaluate the impact of polystyrene‐MXene (PSMX) passivation on the structural, optical, electronic, and PV properties of perovskite films and devices. This work not only demonstrates the feasibility of integrating 2D materials into PSCs but also provides strong evidence that integrating conductive 2D materials with hydrophobic polymers can be an effective strategy to achieve a highly efficient surface passivation layer for PSCs.

## 2 Results and Discussion

**FIGURE 1:**
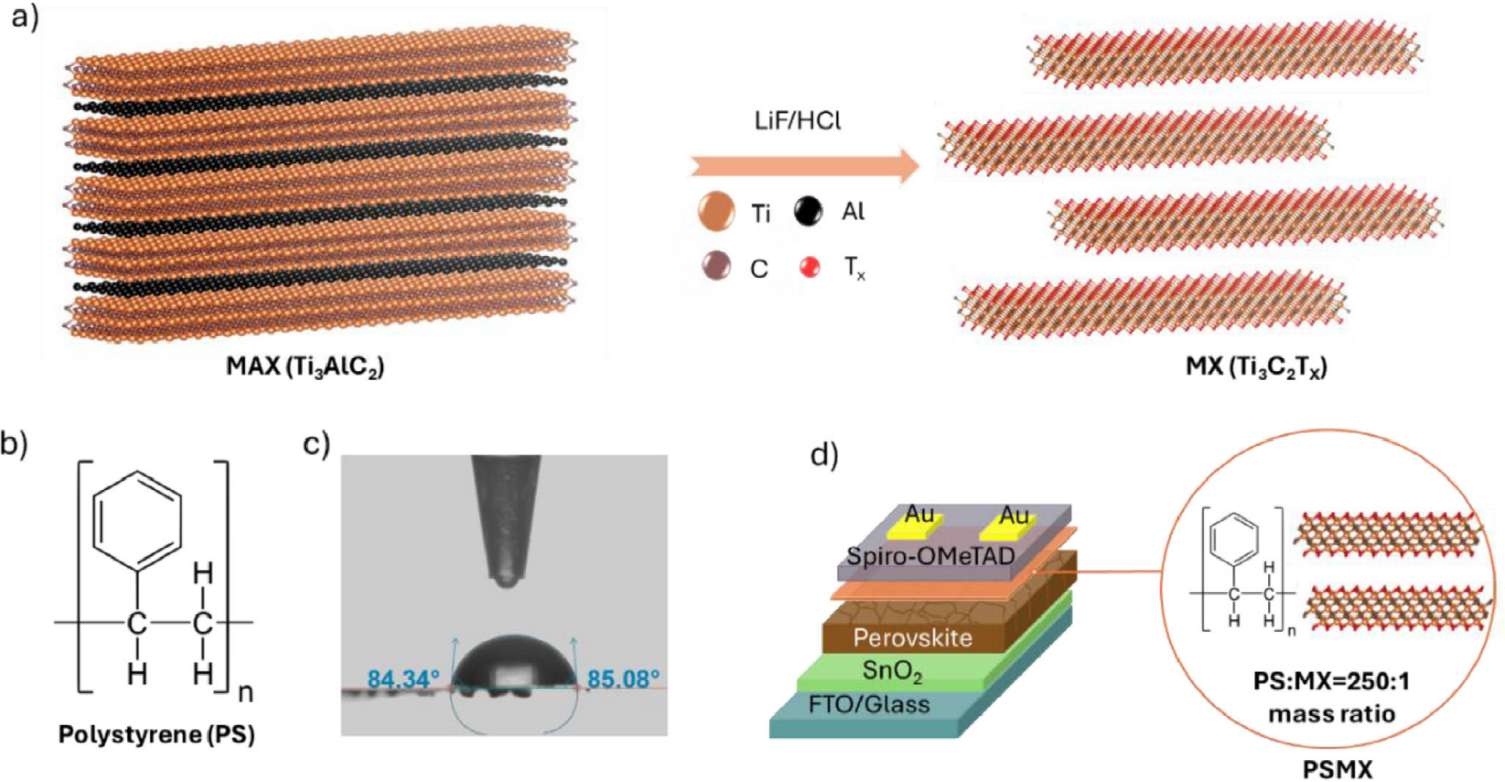
(a) Schematic illustration for the synthesis of few‐layer Ti_3_C_2_T_x_ (MXene). (b) Chemical formula of the PS. (c) Contact angle measurement (water droplet) on a PS film. (d) A full device structure with PSMX passivation layer and (e) the corresponding cross‐sectional SEM image.

A detailed experimental procedure is provided in the Supporting Information. Briefly, Ti_3_C_2_T_x_ sheets were produced from Ti_3_AlC_2_ (MAX phase) according to a traditional minimally intensive layer delamination (MILD) method, as shown in Figure 1a [50]. To obtain Ti_3_C_2_T_x_ aqueous dispersion, the bulk Ti_3_AlC_2_ MAX powder was etched in a LiF and HCl solution by stirring for 24 h at room temperature. After etching, the mixture was washed with deionized (DI) water and centrifuged repeatedly until the pH of the supernatant reached a value of ∼ 6, followed by dispersing in DI water by shaking to obtain an aqueous dispersion of Ti_3_C_2_T_x_ sheets. Then, the as‐synthesized Ti_3_C_2_T_x_ sheets were incorporated with hydrophobic polymer PS with different mass ratios (Figure 1b,c). Subsequently, conventional n–i–p PSCs were fabricated with a layered structure of FTO/SnO_2_/α‐FAPbI_3_/Spiro‐OMeTAD/Au, where the passivation layers were deposited on top of the α‐FAPbI_3_ perovskite. The perovskite layer (reference) was passivated with PEAI, PS, and PSMX (Figure 1d). Structural and morphological characteristics of the films were examined using a range of spectroscopic and microscopic techniques. We first optimized the PS concentration by varying the MXene and PS concentrations, and their detailed results are presented in the PV performance section.

**FIGURE 2:**
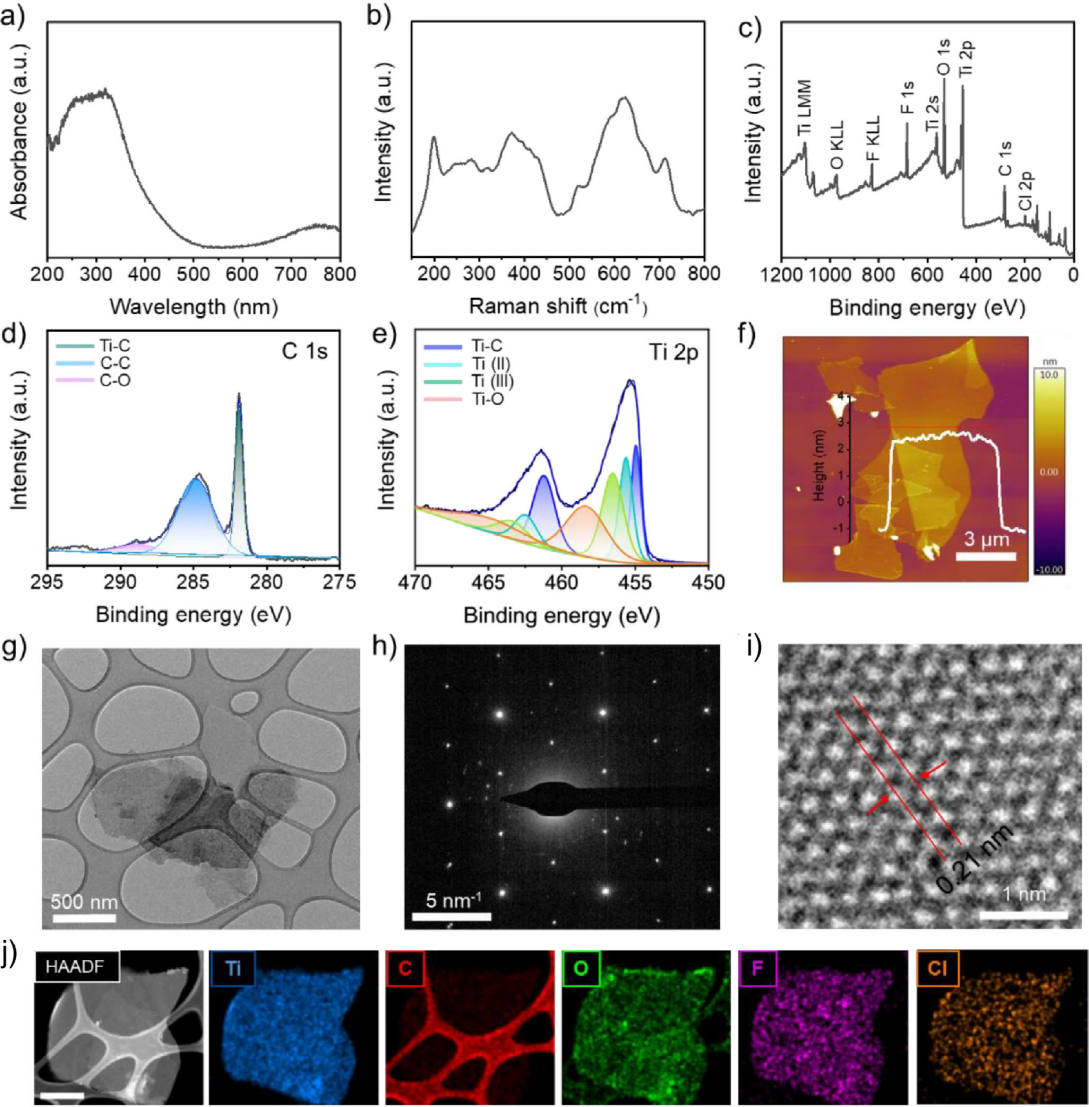
(a) UV–vis and (b) Raman spectrum, (c) XPS survey scan, HR XPS spectra (d) C 1s region, (e) Ti 2p region, and (f) AFM image of the Ti_3_C_2_T_x_. (g) TEM image, (h) SAED pattern, (i) HRTEM image (scale bar: 1 nm), (j) HAADF‐STEM image (scale bar: 400 nm), and the corresponding EDX elemental mapping images of Ti_3_C_2_T_x_ flakes.

The as‐prepared MXene dispersion was studied using Raman spectroscopy and UV–vis spectroscopy. Three main peaks of Ti_3_C_2_T_x_ at wavelengths of 260, 310, and 770 nm were observed from the UV–vis spectrum (Figure 2a) [50, 51, 52]. As shown in Figure 2b, the Raman peaks centered at around 200, 282, 385, 583, and 620 cm^−1^ are the characteristic peaks of Ti_3_C_2_T_x_ MXene, which is consistent with previous literature [43, 53]. The Raman peak at 727 cm^−1^ can be assigned to the coexistence of multiple surface termination groups that have an effect on phonon dispersion of MXene [53, 54]. To confirm the successful synthesis of Ti_3_C_2_T_x_, X‐ray photoelectron spectroscopy (XPS) was performed. Figure 2c shows the survey scan XPS spectrum of the Ti_3_C_2_T_x_ sheets, revealing the presence of Ti, C, F, O, and Cl elements. Besides the Ti, C, and O elements, the existence of F and Cl elements is due to the F and Cl containing surface termination groups caused by the etchant (LiF‐HCl). Moreover, the high‐resolution (HR) XPS spectra of the C 1s (Figure 2d) and Ti 2p (Figure 2e) core levels show the strong Ti–C bonding and a very small peak at around 459 eV assigned to oxidized species of Ti_,_ confirming the successful preparation of Ti_3_C_2_T_x_ flakes. The morphological features of the MXene were examined using atomic force microscopy (AFM) and transmission electron microscopy (TEM). As shown in Figure 2f, the height profile measurement from the AFM image showed that the thickness of our Ti_3_C_2_T_x_ flakes is around 3.5 nm, which is a typical value for few‐layer MXene nanosheets prepared using a MILD method. [50, 55] It can be observed from the TEM image in Figures 2g and 2h, the Ti_3_C_2_T_x_ nanosheets are ultrathin and transparent with a single‐crystal structure. The lateral size of our Ti_3_C_2_T_x_ nanosheets was around 2 µm. As shown in Figure 2i, the lattice fringe of our Ti_3_C_2_T_x_ nanosheets was measured to be 0.21 nm, which is consistent with previous studies [56]. Figure 2j depicts the high‐angle annular dark‐field‐scanning transmission electron microscopy (HAADF‐STEM) image and the corresponding Energy‐dispersive X‐ray (EDX) elemental mapping images of the Ti_3_C_2_T_x_ sheets, indicating the highly uniform distribution of all elements with rich C, O, F, and Cl containing termination groups.

**FIGURE 3:**
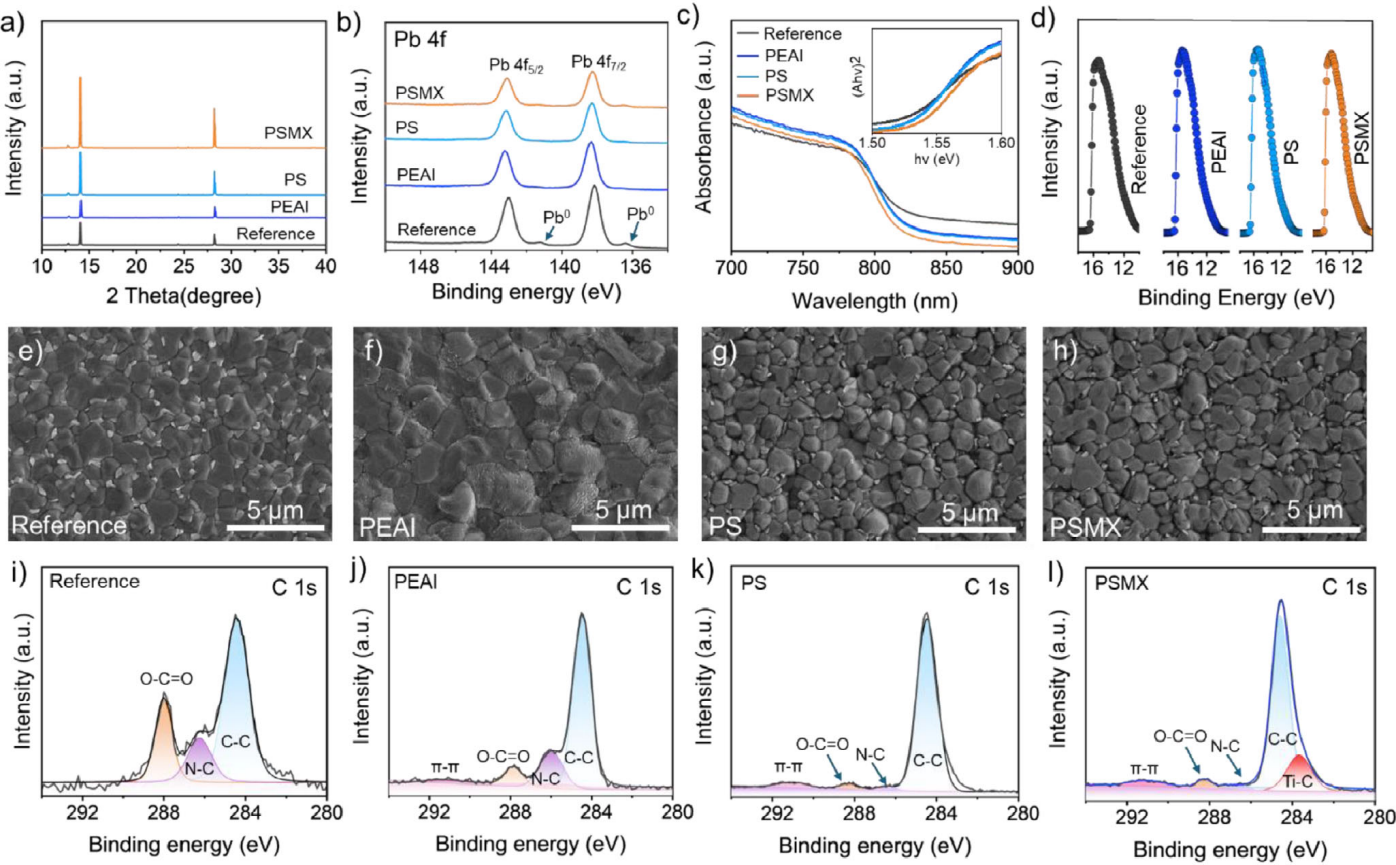
(a) XRD patterns, (b) HR XPS Pb 4f spectra, (c) UV–vis spectra (inset Tauc plot), and (d) UPS spectra of the reference (no passivation), PEAI, PS, and PSMX passivated perovskite thin films. Top‐view SEM images of (e) reference, (f) PEAI, (g) PS, and (h) PSMX passivated perovskite films. HR XPS C 1s spectra of (i) reference, (j) PEAI, (k) PS, and (l) PSMX passivated perovskite films.

The fabricated perovskite thin films with different passivation layers were characterized using X‐ray diffraction (XRD) and XPS. Figure 3a compares the XRD patterns of perovskite films without (reference α‐FAPbI_3_) and with PEAI (α‐FAPbI_3_/PEAI), PS (α‐FAPbI_3_/PS), and PSMX (α‐FAPbI_3_/PSMX). All four samples displayed strong diffraction peaks at 14.1° and 28.2°, corresponding to the (001) and (002) planes of α‐FAPbI_3_, respectively, which are in good agreement with previous literature [42, 57, 58]. Only a negligible amount of PbI_2_ was detected in all samples, indicating that the surface treatments do not adversely affect perovskite crystallization. To confirm the effective surface passivation, XPS measurements were carried out on the perovskite films before and after passivation with PEAI, PS, and PSMX. All four perovskite films displayed comparable XPS survey scan (Figure S1), evidencing the presence of all expected elements. It can be seen from the HR XPS *4f* core level spectra in Figure 3b that the peaks can be deconvoluted into a main doublet with a splitting of ∼4.9 eV. These characteristics peaks correspond to Pb 4*f*_7/2_ and Pb 4*f*_5/2_ doublet associated with Pb‐halide bonding. However, two small peaks of the metallic Pb^0^ at binding energies of 141.2 and 136.4 eV were observed only in the untreated reference film, suggesting that the surface iodine vacancies have been removed after surface passivation.

To elucidate the impact of surface passivation on the perovskite films, we conducted UV–vis absorption and ultraviolet photoelectron spectroscopy (UPS) measurements. As shown in Figure 3c, the optical absorption profiles remain similar across all four samples. The calculated bandgap of the reference film was 1.52 eV, which is in good agreement with α‐FAPbI_3_ values reported in previous studies [58, 59]. The passivated films showed a slight bandgap widening compared to the reference film (Figure 3c inset), with values of approximately 1.53 eV. The energy level alignments of surface‐treated and untreated samples were acquired from both high‐energy (Figure 3d) and low‐energy UPS data (Figure S2). Notably, we noticed slight upshifts in the valence band maximum (VBM) and conduction band minimum (CBM) of all passivated perovskite films relative to the reference, indicating improved energy level alignment with the hole‐transport material (spiro‐OMeTAD). Among the treatments, PS showed the largest upshift. These results suggest that PSMX favorably tunes the interfacial energetics, showing great promise for PSCs (Figure S3) [43].

Figure 3e–h show the top‐view scanning electron microscopy (SEM) images of the perovskite films without and with PEAI, PS, and PSMX surface treatment. While noticeable differences can be observed from these SEM images, all samples showed well‐organized, pinhole‐free grains. Notably, the PEAI‐treated film has a larger average grain size (∼2 µm, Figure 3f) as compared to the other perovskite films (∼1.8 µm). The PS (Figure 3g) and PSMX coated films display (Figure 3h) uniform grain‐size distributions significantly reduced amount of PbI_2_ on the surface in comparison to the reference film (Figure 3e). The treated films (PEAI, PS, and PSMX) exhibit more textured surfaces, resembling 2D/3D perovskite heterostructures, whereas the reference film appears relatively flat [24, 25]. As shown in Figure 3e, bright contrast at the grain boundaries indicates the presence of PbI_2_, due to its higher average atomic number. Prior studies indicate that excess PbI_2_ can accelerate perovskite degradation, leading to rapid losses in PV performance, which we address in the stability section [60, 61]. After spin‐coating PEAI (Figure 3f), the surface PbI_2_ is markedly reduced, consistent with the formation of a capping layer. Notably, PS and PSMX treatments produce a similar effect, likewise promoting a capping layer on the perovskite surface. Additionally, atomic force microscopy (AFM) was performed to evaluate the surface roughness of the films. The root mean square (RMS) roughness values for the reference, PEAI, PS, and PSMX films were measured to be 34.14, 27.28, 30.70, and 28.04 nm, respectively (Figure S4 (top)).

Further, we have conducted HR XPS C *1s* spectroscopy measurements to confirm the treatment of passivation layers (PEAI, PS, and PSMX) on the perovskite films (Figure 3i–l). The C 1s spectra can be deconvoluted into characteristics C─C, C─N, and C═O peaks located at 284.4, 286.3, and 287.9 eV, respectively. The reference film shows a noticeably higher intensity of C═O peak located at 287.9 eV as compared to the surface‐treated films (Figure 3i), indicating greater surface oxidation likely introduced during the sample transfer into a XPS chamber. In contrast, all PEAI, PS, and PSMX treated perovskite films exhibited reduced C═O peak intensities while π−π bonding peak emerged at ∼291.0 eV, which is consitent with the presence of phenyl functional group [10, 24]. Notably, PS and PSMX passivated films displayed only very weak C═O peak, suggesting that PS‐based passivation provides better resistance to oxygen/moisture as compared to the PEAI passivation (Figure 3j–l). The HR C 1s spectrum of the PSMX film was carefully deconvoluted in Figure 3l. A weak component centered at ∼283 eV was identified, which can be attributed to the Ti─C bond originating from Ti_3_C_2_T_x_. Owing to the low MXene loading, this peak appears with low intensity and partially overlaps with adjacent carbon‐related signals, resulting in its limited visibility in the raw spectrum. Furthermore, according to the Pb 4*f* and I 3*d* core‐level energy spectra, the Pb: I ratio for the reference perovskite film was calculated to be 1:2.17, while PEAI surface treatment increased this value to 1:2.72, suggesting reduced iodine vacancies in the perovskite (Figure S5). The Pb: I ratio of PS and PSMX passivated perovskite films were estimated to be 1:2.23 and 1:2.36, respectively, reflecting a moderate increase in the iodine content as compared to the reference sample. These results suggest that these passivation layers help to mitigate iodine volatility and migration, likely through surface passivation by the PS‐based molecules.

**FIGURE 4:**
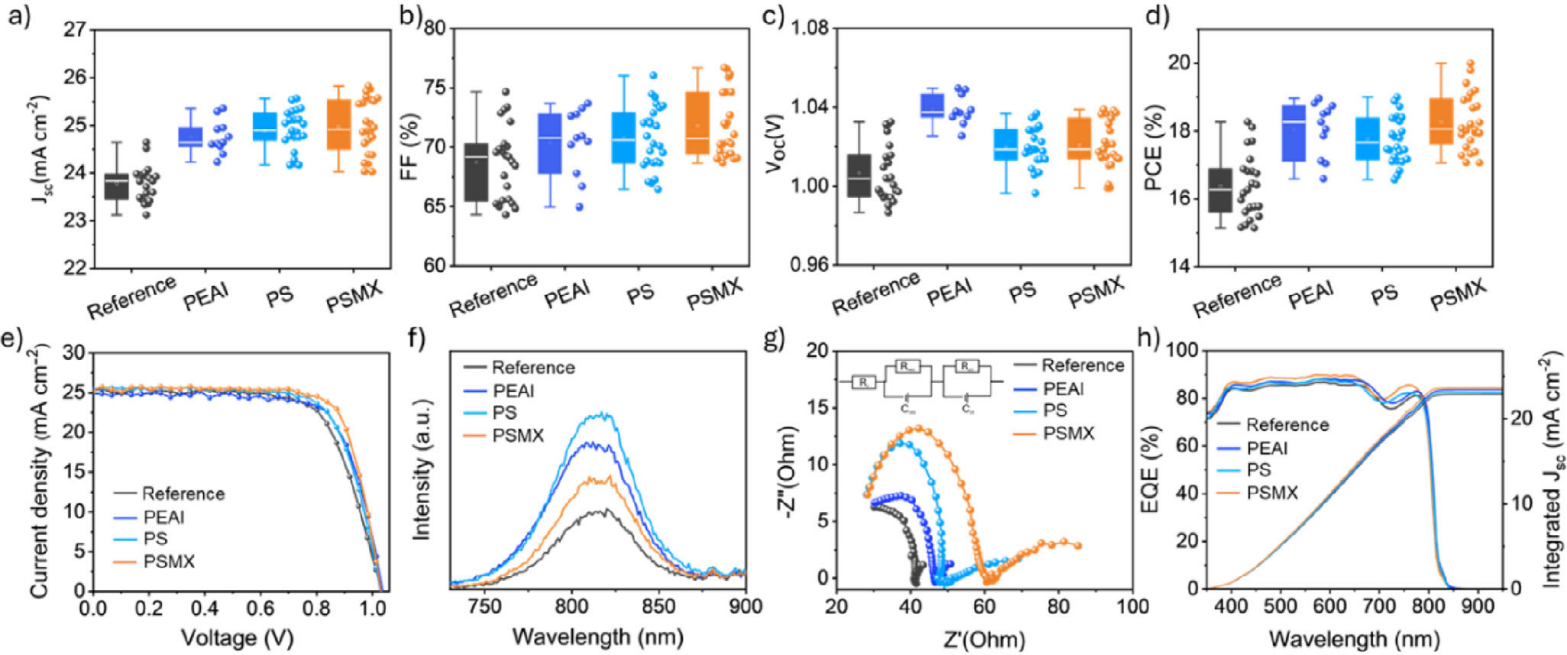
Statistical distribution of the PV parameters (a) J_SC_, (b) FF, (c) V_OC,_ and (d) PCE of reference, PEAI, PS, and PSMX passivated PSCs. (e) *J*–*V* curves of the best performing devices. (f) PL spectra of perovskite films with different passivations. (g) EIS Nyquist plots and (h) EQE spectra of the reference, PEAI, PS, and PSMX passivated PSCs.

To assess the impact of PS and PSMX passivation on the device performance, n–i–p PSCs with the following architecture were fabricated: FTO/SnO_2_/FAPbI_3_/passivation layer/Spiro‐OMeTAD/Au. We first optimized the PS concentration to ensure an efficient charge transport while achieving an effective passivation layer. Too thick a PS layer is expected to act as an insulating layer, blocking charge transport. Figure S6 summarizes the PV parameters of devices treated with PS at 1, 2.5, 5, and 7.5 mg mL^−1^ concentrations. The PCE increased with PS (molecular weight ∼30 000) concentration up to 2.5 mg mL^−1^, at which the highest efficiency was achieved. Further increasing to 5 mg mL^−1^ concentration led to a decline in PCE, driven by reductions in fill factor (FF) and short‐circuit current (J_SC_). This decline is attributed to the thick PS layer acting as a barrier for hole injection into the HTL. Based on these results, 2.5 mg mL^−1^ was selected as the optimal PS concentration for subsequent device fabrication. To examine the effect of Ti_3_C_2_T_x_ incorporation, PSCs were fabricated using PSMX solutions with PS: MX mass ratios of 500:1, 375:1, 250:1, and 125:1. All dispersions were sonicated in chlorobenzene for 30 min to reduce the aggregation of the Ti_3_C_2_T_x_ nanosheets before spin coating onto the perovskite layers (Figure S7). When adding Ti_3_C_2_T_x_ in the PS solution, the PCE increased until the ratio of 1:250 and then started to decline with further increasing the Ti_3_C_2_T_x_ contents (Figure S8). Figure S9 presents a cross‐sectional SEM image of the PSMX passivated full device with a mass ratio of 250:1 (PS: MX), revealing the layered architecture of FTO/SnO_2_/α‐FAPbI_3_/PSMX/Spiro‐OMeTAD/Au. The ∼30 nm SnO_2_ layer was formed on the FTO substrate, while ∼20 nm PSMX passivated the 500 nm thick perovskite with a flat and smooth contact with a 160 nm Spiro‐OMeTAD layer. Finally, a 75 nm gold electrode was thermally evaporated. Figure 4a–d summarize the PV parameters of the reference (without passivation), PEAI, PS, and PSMX (PS250‐MX) passivated PSCs such as J_SC_, open‐circuit voltage (V_OC_), FF, and PCE. The photocurrent‐voltage (J–V) curves of the best‐performing devices are displayed in Figure 4e. It can also be clearly observed that the devices with PSMX passivation showed excellent reproducibility as compared to the reference, PEAI, and PS only passivated devices. The best performing reference device showed a maximum PCE of 18.27% with J_SC_ of 24.24 mA cm^−2^, V_OC_ of 1.03 V, and FF of 73.17%, while the devices using PEAI as a passivation layer delivered a slightly improved PCE of 18.96% with the measured J_SC_ value of 24.95 mA cm^−2^, V_OC_ of 1.04 V, and FF of 73.33%. In comparison, the devices using PS as a passivation layer delivered a PCE of 19% with the measured J_SC_ value of 25.56 mA cm^−2^, V_OC_ of 1.03 V, and FF of 72.19%. These results suggest that the PS treatment with its optimal concentration (2.5 mg mL^−1^) can be as competitive passivation as PEAI for PSCs. Furthermore, the devices using PSMX as a passivation layer achieved a PCE of 20.01% with J_SC_ of 25.54 mA cm^−2^, V_OC_ of 1.03 V, and 75.90%. The considerable PCE enhancement primarily originated from the improved J_SC_ and FF when PS is mixed with MXene for passivation. This can be attributed to the incorporation of Ti_3_C_2_T_x_ MXene, which reduces the resistance of the PS film and enhances interfacial charge transport. This effect is attributed to dispersed conductive MXene within the PS, which facilitate localized charge transfer and improve carrier extraction, rather than the formation of a continuous conductive network. To support this, PEAI, PS, and PSMX were spin‐coated onto bare glass substrates, and their sheet resistances were measured (Figure S10). The sheet resistance of the PS film was measured to be 17.5 MΩ sq^−^^1^, while the PS: MXene film (250:1 mass ratio) exhibited a significantly lower value of 1.2 MΩ sq^−^^1^. These results indicate that the incorporation of a small amount of MXene significantly improves the effective conductivity of the PS film, likely through localized conductive pathways and interfacial effects associated with dispersed MXene domains. Moreover, the use of PSMX leads to a favourable interfacial band shift, which provides suitable energy level alignment between the perovskite absorber to the hole‐transporting layer as compared to PS (Figure S11). However, an excessive amount of Ti_3_C_2_T_x_ can lead to aggregation in the PS, which results in reduced PV performances.

We employed steady‐state photoluminescence (PL) and time‐resolved PL (TRPL) measurements to evaluate non‐radiative recombination in our surface passivated perovskite films. As shown in Figure 4f, the PL intensity of all passivated films was higher than that of the untreated reference perovskite/glass film. Consistent with previous reports, PEAI treatment (without annealing) increased the PL intensity, indicating effective suppression of non‐radiative recombination via defect passivation [10]. Notably, the PS passivated film exhibited even higher PL intensity than the PEAI passivated perovskite film under 355 nm excitation applied through the glass side, suggesting that the hydrophobic PS layer acts as effective surface passivation while also shielding the perovskite layer from moisture. Moreover, Li et al. previously showed that a significant intensity increase in PL spectra was observed from the concentration ranging from 1 to 5 mg mL^−1^, followed by a clear decrease when a higher concentration of PS was used [62]. Interestingly, in our work, introducing Ti_3_C_2_T_x_ into the PS layer (PSMX) reduced the PL intensity relative to the PS only layer, but it still remains higher than that of the reference film. This intensity reduction when MXene is added in the PS layer can be attributed to the high electrical conductivity of Ti_3_C_2_T_x_, which facilitates interfacial charge transfer and thus promotes hole transport to the Spiro‐OMeTAD. Moreover, time‐correlated single‐photon counting was used to acquire a more quantitative understanding of charge kinetics. The corresponding average bi‐exponential lifetimes of PS and PSMX passivated films were calculated from the PL decay curves, as illustrated in Figure S12. The PS passivated film showed a longer lifetime (13.5 ns) than the PSMX (9.8 ns) treated film. This result is in agreement with the PL measurements. Importantly, PS still acts as a defect passivation layer in PSMX, indicating the optimized PS: MXene can not only effectively passivate surface defects but also imroves charge transfer kinetics of PSCs. This behaviour aligns with the PV parameters, where enhanced charge extraction resulted in increased J_SC_ and FF in PSMX‐treated PSCs.

To investigate the charge transfer and recombination processes of surface passivated PSCs, we performed electrochemical impedance spectroscopy (EIS) measurements and analyzed the data using ZSimpView. Figure 4g presents the Nyquist plots of the devices without and with surface passivation. Two typical semicircles were observed for all Nyquist plots in the measured frequency range. The circuits consist of a series resistance (R_S_) and a charge recombination resistance (R_rec_) with a layer capacitance (C) [62, 63, 64]. The R_S_ mainly occurred at the interface of FTO and the electron‐transporting layer, while R_rec_ is associated with the perovskite/Spiro‐OMeTAD interface. The low‐frequency semicircle is mainly related to slow ion relaxation and diffusion process in the perovskite layer as fitted using an equivalent circuit diagram presented in Figure 4g inset. The reference device showed the lowest R_rec_ of 130.5 ohm cm^−2^, indicating increased charge recombination. Meanwhile, the R_rec_ values of PEAI and PS passivated devices were measured to be 290.7 and 250.8 ohm cm^−2^, respectively. This suggests that surface passivation reduced the charge recombination loss as it increased R_rec_. The PSMX passivated PSC exhibited an increased R_rec_ (326.0 ohm cm^−2^) as compared to the PS passivated device, suggesting further suppression of interfacial recombination and improved charge transfer between the perovskite film and Spiro‐O‐MeTAD, likely facilitated by the excellent electrical conductivity of the MXene component. To further examine the charge extraction characteristics of the PS and PSMX passivated devices, *J*–*V* measurements were conducted in both reverse (+1.2 V to −0.1 V) and forward (−0.1 V to +1.2 V) scan directions to evaluate the hysteresis behaviour and charge‐extraction capability of the PS and PSMX passivated devices. The comparison between the PS (Figure S13a) and the PSMX‐based (Figure S13b) reveals that incorporating the MXene with PS significantly suppresses hysteresis relative to the device with only PS. To quantitatively assess this behaviour, the hysteresis index (HI) was calculated according to Equation 1 [42].

(1) $$\mathrm{HI}\;=\frac{\;\mathrm{PC}E_{\mathrm{reverse}}-\mathrm{PC}E_{\mathrm{forward}}}{\mathrm{PC}E_{\mathrm{reverse}}}\;$$

The HI was markedly reduced from 0.179 to 0.120 in the presence of MXene, indicating improved interfacial contact between the perovskite and Spiro‐OMeTAD. The observed suppression of hysteresis may also be attributed to the reduced ion migration within the perovskite layer in the presence of PSMX. Collectively, these findings confirm the effective hole‐transporting capability and interfacial modulation provided by the PSMX passivation layer. External quantum efficiency (EQE) measurements were conducted and the spectra are shown in Figure 4h. All PSCs displayed excellent light absorption and conversion efficiencies at wavelengths ranging from 350 to 850 nm. The J_sc_ values integrated from the EQE spectra were 22.8, 23.4, 23.1, and 23.6 mAcm^−2^ for the reference, PEAI, PS, and PSMX passivated devices, respectively. These values corroborate within the statistical distribution of the corresponding measured J_sc_ values.

**FIGURE 5:**
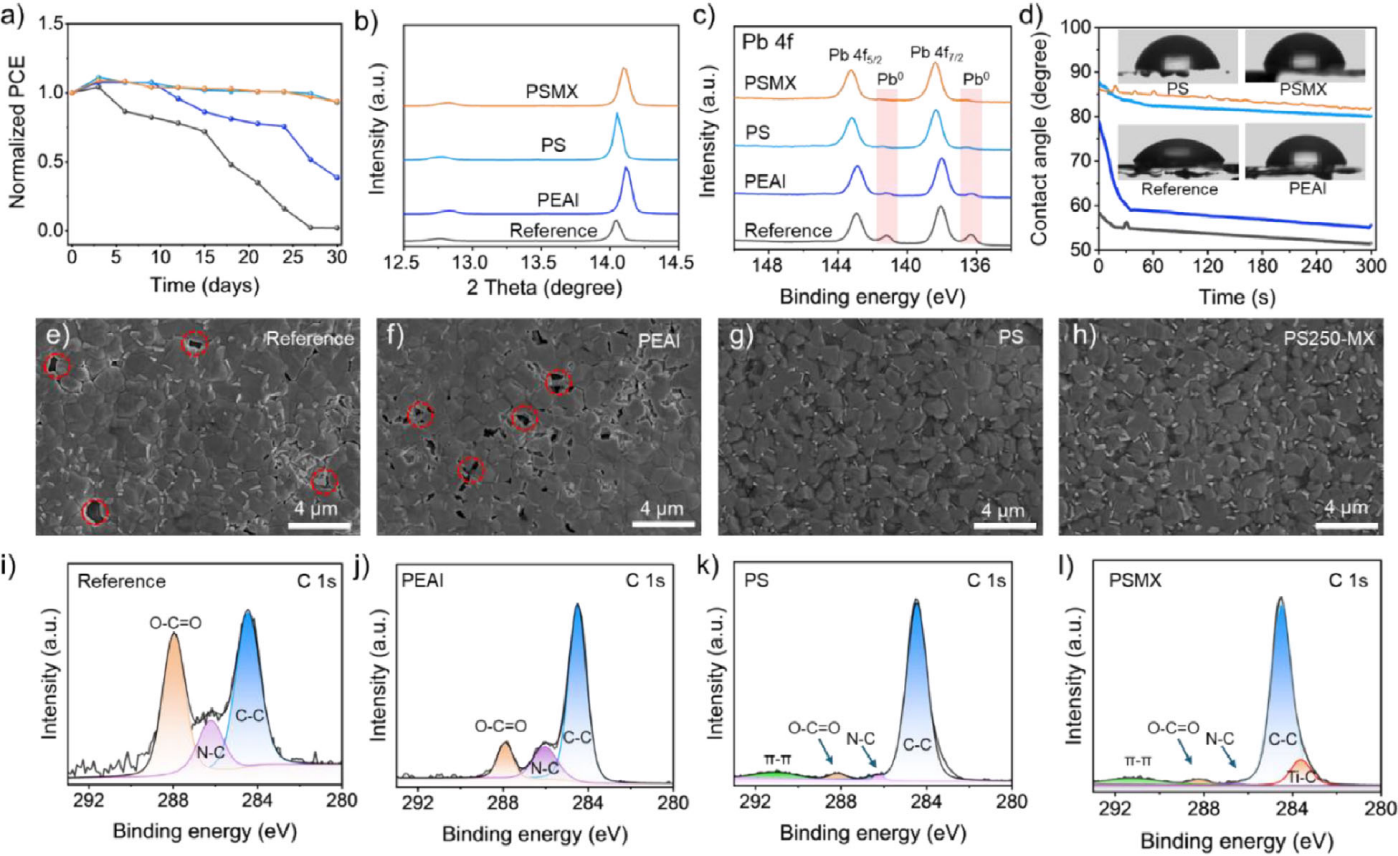
(a) Stability test of unencapsulated reference, PEAI, PS, and PSMX passivated PSCs aged in ambient air with 40–50% RH for 30 days. (b) XRD patterns, (c) XPS HR Pb 4f spectra of aged films at ∼75% RH for 2 h. (d) Average contact angle measurements of the reference, PEAI, PS, and PSMX passivated perovskite films over 300 s. The inset shows the photograph of contact angle measurements of the water droplet at 0 s. (e–h) Top view SEM images and (i–l) HR XPS C1s spectra of aged reference, PEAI, PS, and PSMX passivated perovskite films in ambient conditions with RH ∼75% for 2 h.

Given the better performance of the PSMX passivated PSCs, we further tested their long‐term stability under ambient conditions in the dark (at a relative humidity (RH) of ∼40%–50% in air) without any encapsulation over 30 days (Figure 5a). In particular, the reference device without passivation retained only 2.05% of its initial PCE after 30 days. Meanwhile, the PEAI passivated device retained 38.55% of its initial efficiency, suggesting improved device stability, which is often observed in literature. The PEAI is widely recognized for enhancing the stability of PSCs through suppressing iodine migration to the Spiro‐O‐MeTAD and mitigating interfacial defects. Importantly, PS and PSMX passivated devices showed similar stability results, retaining 92.96% and 93.63% of its initial PCE, respectively. The improved stability of PS and PSMX devices is attributed strongly to the hydrophobic nature of PS, preventing perovskite from moisture in ambient conditions. Furthermore, we investigated the chemical composition of perovskite films without and with a passivation layer. After aging perovskite films in ∼75% RH conditions for 2 h, we conducted XRD measurements. As shown in Figure 5b, all four aged α‐FAPbI_3_ films with different passivation retained the characteristic XRD patterns as observed in the fresh samples at near 14.1°. However, a more evident additional peak emerged at 11.8° in the reference film in Figure S14, corresponding to the non‐photoactive δ‐FAPbI_3_ phase [65, 66]. In the aged PS‐, PSMX‐, and PEAI‐passivated films, the diffraction feature near 11.8° is negligible, suggesting that only a minimal amount of δ‐FAPbI_3_ may have formed during fabrication. After aging, the weak peak observed in the PS and PSMX samples is therefore attributed to a minor buried δ‐phase within the bulk, while the surface remains effectively passivated and stable, consistent with their superior device performance compared to the PEAI‐treated films (Figure S14). Furthermore, we conducted HR XPS Pb 4f measurements on aged perovskite films without and with PEAI, PS, and PSMX passivation after storing in ambient conditions for 2 h. As compared to the fresh sample, the aged films exhibited metallic Pb^0^ peaks (Figure 5c). The calculated Pb^0^/Pb^2+^ ratios for the reference, PEAI, PS, and PSMX passivated perovskite films were 26.2%, 12.0%, 7.2% and 6.6%, respectively.

To confirm the hydrophobic nature of PS‐based layers on top of the perovskite layer, contact angles of water droplets on the reference, PEAI, PS, and PSMX passivated films were measured over 300 s (see Figure 5d). It can be seen from the inset of Figure 5d that the initial contact angle of each film was 58.9°, 79.5°, 87.8° and 86.5° for reference, PEAI, PS, and PSMX, respectively. Unlike reference and/or PEAI‐treated perovskite films, the contact angles of PS and PSMX‐treated films did not decrease significantly after 300 s of continuous measurement, supporting our long‐term stability test. Moreover, top‐view SEM images of aged films revealed that morphological changes were observed near grain boundaries, including noticeable pinholes in the reference and PEAI passivated films (Figure 5e,f). In contrast, PS and PSMX passivated films remained unchanged due to their outstanding hydrophobicity (in Figure 5g,h). Low‐magnification SEM images are also provided in Figure S15, showing no evidence of pinholes or grain boundary separation in the PS and PSMX passivated perovskite films, corroborating the better stability of perovskite films. As shown in Figure S4 (bottom), the RMS values after aging were 58.17, 40.92, 30.94, and 28.92 nm for the reference, PEAI, PS, and PSMX treated films, respectively. These results suggest that the PS and PSMX passivated perovskite films undergo substantially less morphological change compared with the reference and PEAI‐treated films after 2 h of aging under ambient conditions at ∼75% RH.

Next, we conducted HR XPS C 1s measurements on our aged samples, as shown in Figure 5i–l. As compared to the fresh reference and PEAI films, the aged reference and PEAI films showed increased C═O intensity at ∼288 eV, suggesting high oxygen adsorption on the perovskite surface during aging in ambient conditions (see Figure 5i,j). Interestingly, the aged PS and PSMX passivated films showed no changes as compared to their fresh samples, still displaying *π*–*π* peak of the phenyl group at ∼291 eV. Notably, the characteristic *π*–*π* peak of the phenyl group in the PEAI‐passivated film disappeared after aging (Figure 5j), consistent with the changes observed in the SEM image (Figure 5f). These results suggest that combining conductive MXene with PS significantly helps to maintain the perovskite structure even in highhumidity conditions.

## 3 Conclusion

In summary, we demonstrated a promising surface passivation strategy that integrates conductive 2D Ti_3_C_2_T_x_ into hydrophobic PS to fabricate stable PSCs. We found that highly conductive Ti_3_C_2_T_x_ nanosheets reduce the resistance of the PS film, which can effectively passivate surface defects and decrease non‐radiative recombination in PSCs. As a result, the devices fabricated with optimized PSMX passivation exhibited >20% efficiencies with significantly enhanced stability. The stability tests showed that both PS and PSMX passivated cells retain 93% of their initial efficiencies, whereas the reference and PEAI passivated devices maintained less than 40% of their initial efficiencies. These results establish that PSMX can serve as an effective passivation or encapsulation layers for enhancing the durability and performance of PSCs.

## Conflicts of Interest

The authors declare no conflicts of interest.

## Supporting information




**Supporting File**: smll73908‐sup‐0001‐SuppMat.docx.

## Data Availability

The data that support the findings of this study are available from the corresponding author upon reasonable request.
