# Supplementary material for: Ti3C2Tx (MXene)‐Polystyrene Based Passivation for Perovskite Solar Cells With Enhanced Stability
Source: Small. 2026 May 24;22(40):e13734. doi: 10.1002/smll.202513734 (PMC13378644; doi:10.1002/smll.202513734)
Supplement: Supplementary file 1 — Supporting File: smll73908‐sup‐0001‐SuppMat.docx. [file SMLL-22-e13734-s001.docx]

Supporting Information

# **Ti_3_C_2_T_x_ (MXene)-Polystyrene Based Passivation for Perovskite Solar Cells with Enhanced Stability**

*Selengesuren Suragtkhuu, Purevlkham Myagmarsereejid, Isaac Etchells, Paul E. Shaw, Yu Lin Zhong, Munkhbayar Batmunkh^*^*

S. Suragtkhuu, P. Myagmarsereejid, Y.L. Zhong, M. Batmunkh

School of Environment and Science, Griffith University, Nathan, Queensland 4111, Australia.
E-mail: [m.batmunkh@griffith.edu.au](mailto:m.batmunkh@griffith.edu.au)

I. Etchells, P.E. Shaw

Centre for Organic Photonics & Electronics, School of Chemistry & Molecular Bioscience, The University of Queensland, Brisbane 4072, Qld 4072, Australia

(“S.S. and P.M. contributed equally to this work.”)

Experimental Section

*Materials*

Unless otherwise stated, all chemicals were obtained from Merck. Pre-patterned fluorine-doped tin oxide (FTO) glass substrates (2.5 cm x 2.5 cm) were purchased from Xin Yan Technology Ltd. Tin oxide (SnO_2_) colloidal solution (15 wt% in DI water) was obtained from Alfa Aesar. Lead iodide (PbI_2_) (ultra dry, 99.999%, metal basis) was purchased from Thermo Fisher. Formamidinium iodide (FAI), phenylethylammonium iodide (PEAI) and methylammonium iodide (MACl) were purchased from Greatcell Solar. 2,2′,7,7′-tetrakis(N,N-di-p-methoxyphenylamine)-9,9′-spirobifluorene (Spiro-OMeTAD) was purchased from Xi’an Yuri Solar Co. Ltd.

*Synthesis of α-formamidinium lead triiodide (α-FAPbI_3_) powder*

The α-phase FAPbI_3_ (α-FAPbI_3_) powder was prepared according to an established literature protocol.^[1]^ The black α-FAPbI_3_ powder was prepared by adding FAI (3.36 g) and PbI_2_ (9 g) into an anhydrous 2-Methoxyethanol (2-ME) (11 mL) in a 50 mL vial. The solution was then heated at 125°C and stirred for 45 min. Then, the black precipitate was filtered using a Whatman filter paper before cooling it to room temperature. The obtained α-FAPbI_3_ was heated at 150 °C for 30 min before being transferred to a vacuum desiccator. After storing in a desiccator overnight, the as-prepared α-FAPbI_3_ powder was then transferred to an N_2_-filled glovebox for storage and further use.

*Synthesis of Ti_3_C_2_T_x_ (MXene)*

Ti_3_C_2_T_x_ (MXene) was prepared using an optimized minimally intensive layer delamination (MILD) procedure.^[2]^ Briefly, 4 g LiF (325 mesh powder, Alfa Aesar, USA) was added to 50 mL of HCl (32 %, 9M) with continuous stirring to obtain a homogeneous solution. After that, bulk Ti_3_AlC_2_ (2.5 g) was crushed using a mortar and pestle and added to the mixture solution (LiF/HCl). This process was conducted at a room temperature (25˚C) for 24 h with a continuous stirring at 400 rpm. The mixture was then washed with deionized (DI) water and centrifuged until the pH exceeded 6. The pellet at the bottom was collected and redispersed in DI water via manual shaking, while the supernatant was discarded. After few cycles of centrifuging, the supernatant was discarded as the flake size was small. The swollen pellet at the bottom was collected and redispersed in DI water through very gentle shaking. The obtained Ti_3_C_2_T_x_ flakes were then freeze-dried and redispersed in chlorobenzene.

*Preparation of PSMX composite*

For the synthesis of PSMX composite, the as-prepared Ti_3_C_2_T_x_ flakes were added into the 2.5 mg mL^-1^ PS solution in chlorobenzene. The weight ratios of PS and MXene were 500:1, 375:1, 250:1 and 125:1. All dispersions were sonicated in chlorobenzene for 30 min to reduce the aggregation of the Ti₃C₂Tₓ nanosheets before spin coating onto the perovskite layers.

*Device fabrication*

FTO substrates were sonicated in Hellmanex III, DI water, acetone, and isopropyl alcohol (isopropanol/IPA) for 15 min each before being treated with ultraviolet (UV) ozone for 10 min. A 30 nm SnO_2_ film was deposited by spin coating diluted SnO_2_ solution (2.67% in water) onto the cleaned FTO at 3,000 rpm for 30 s, followed by annealing at 150° C for 30 min in air. After cooling to room temperature, the SnO_2_/FTO substrates were again treated with UV ozone for 10 min before being transferred to N_2_ glovebox. The perovskite solution was prepared by dissolving 1.4 M FAPbI_3_ black powder with 35 mol% MACl, and 3.8 mol% methylenediammonium chloride (MDACl_2_) in a mixed solvent (dimethylformamide (DMF) and dimethyl sulfoxide (DMSO) (8:1 v/v)). The perovskite solution was deposited onto the SnO_2_ film by spin coating at 1,000 rpm for 10 s and 5,000 rpm for 30 s. During the spinning, 1 mL diethyl ether (DEE) was dripped onto the substrate after 10 s at 5,000 rpm. The as-deposited perovskite films were annealed at 125°C for 60 min in air with controlled humidity of 25%–40%. Subsequently, the substrates were transferred to the glovebox. For PEAI based devices, perovskite layers were passivated using a 15 mM PEAI solution in IPA at 5,000 rpm for 30 s. For the fabrication of PS and PSMX passivated perovskite films, PS and PSMX solution (in chlorobenzene) was deposited on top of the perovskite films by spin coating at 4,000 rpm for 20 s, followed by annealing at 100˚C for 2 min. A hole-transporting material (HTM), Spiro-OMeTAD in chlorobenzene, was deposited onto the passivated and non-passivated perovskite layers by spin coating at 3,000 rpm for 30 s. The HTM solution was prepared by mixing 90 mg Spiro-OMeTAD, 39 µL 4-tert-butylpyridine (4-tBP, 98%), and 23 µL of a stock solution of lithium bis(trifluoromethylsulphonyl)imide (Li-TFSI, 99.95%) in acetonitrile (520 mg mL^–1^), in 1 mL chlorobenzene. The devices were left inside a desiccator overnight to improve the conductivity of the Spiro-OMeTAD. Finally, a 75 nm thick gold (Au) electrode was deposited using a thermal evaporator. The active area of the device was 0.1010 cm^2^.

*Materials characterizations*

A field-emission-scanning electron microscope (JOEL 7100F) was to obtain the scanning electron microscopy (SEM) images. The optical absorbance data were collected using a UV–vis spectrometer (Agilent UV/Vis 8454). Raman spectra were acquired using a WITec alpha300 RA + S with a 40× objective. Data from transmission electron microscopy (HT7700, TEM) was acquired using 120 kV beam energy. X-ray photoelectron spectroscopy (XPS) data were acquired using a Kratos Axis ULTRA X-ray Photoelectron Spectrometer incorporating a 165 mm hemispherical electron energy analyzer. The incident radiation was monochromatic Al Kα X-rays (1486.6 eV) at 225 W (15 kV, 15 mA). Base pressure in the analysis chamber was 1.0 × 10^−9^ torr and during sample analysis 1.0 × 10^−8^ torr. Atomic force microscopy (AFM) was performed in air using Asylum Research Cypher S with Asylum Research software, operating in standard tapping mode configuration using AIR cantilever holder. TEM measurements were performed using a FEI Titan Themis. Scanning transmission electron microscopy (STEM) imaging and Energy-Dispersive X-ray (EDX) elemental mapping were carried out also on a FEI Titan Themis S-TEM instrument. The STEM probe was aberration-corrected, enabling sub-angstrom spatial resolution, and HAADF images were obtained.

*Film and device characterizations*

X-ray photoelectron spectroscopy (XPS) was obtained using a Kratos Axis Supra^+^ using an Al Kα (λ=1486.6 eV) and He I (λ=21.22 eV) source, respectively. The crystal structures of perovskite films were recorded using a X-ray diffraction (the Bragg-Bentono geometry) on a Rigaku Smartlab diffractometer equipped with a 9 kW Cu rotating anode operating at 45 kV and 200 mA with a scanning range from 5º to 55º (2*θ*). UV-vis spectra were recorded on an Agilent UV/Vis 8454-visible spectrophotometer. J-V characteristics were carried out in air using a Ossila J-V test system under 1 sun illumination (AM 1.5 G, 100 mW cm^-2^) with a scan rate of 125 mV s^-1^. The illumination intensity was 100 mW cm^-2^ from the solar simulator (Sun 2000, Abet Technologies). The solar simulator was calibrated against a National Renewable Energy Laboratory (NREL) certified standard 2 cm × 2 cm Si photodiode. All measurements were performed at room temperature without encapsulation. Steady-state PL spectra were conducted using an Edinburgh Instruments FS5 fluorometer with an excitation wavelength of 450 nm. Time-resolved PL decays were scanned using a Fluorolog-3 with time-correlated single photon counting capability. The excitation source was a light-emitting diode emitting at 441 nm pulsed at 1 MHz with a pulse width of 1.2 ns, and the PL decays were measured at 770 nm. Electrochemical impedance spectroscopy (EIS) measurements were carried out a potentiostatic excitation applied frequencies between 10^-1^ and 10^6^ Hz under illumination using Gamry electrochemical workstation (Interface 1010E). Spectra were fitted using a ZView software. Contact angle measurements carried out using an optical tensiometer (Theta Flex, Biolin Scientific, Finland) and drop size was 5 µL recorded contact duration for 300 s.


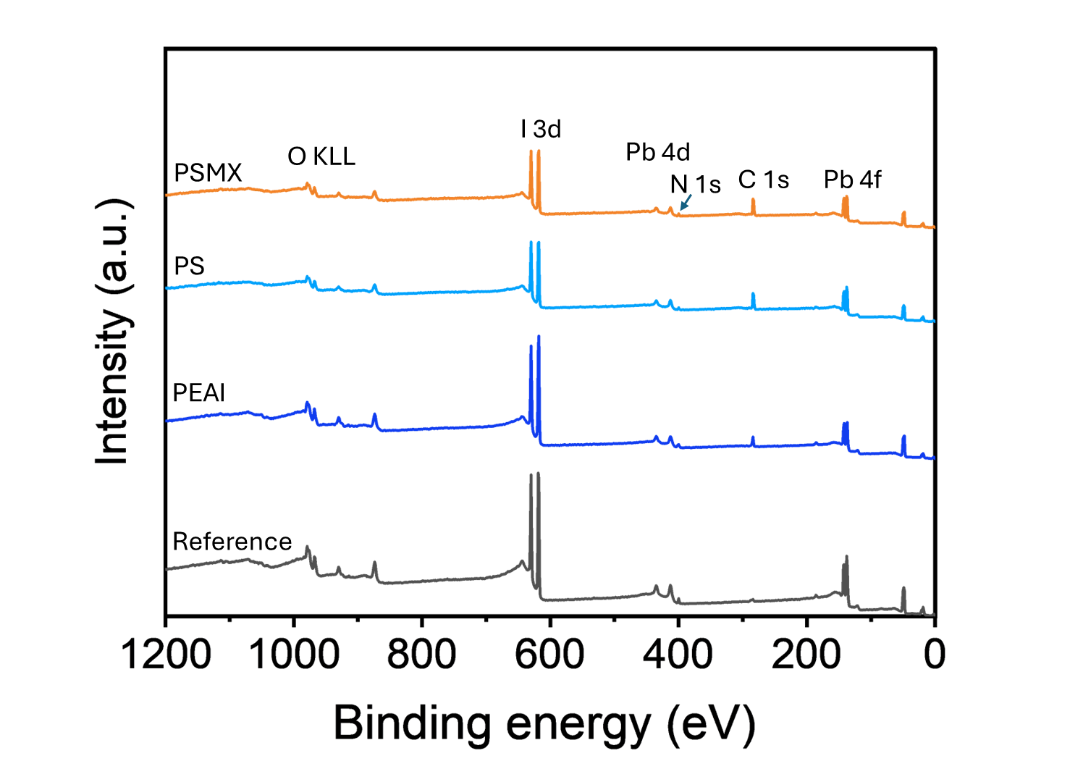


**Figure S1.** XPS survey scan of reference, PEAI, PS and PSMX passivated perovskite film.


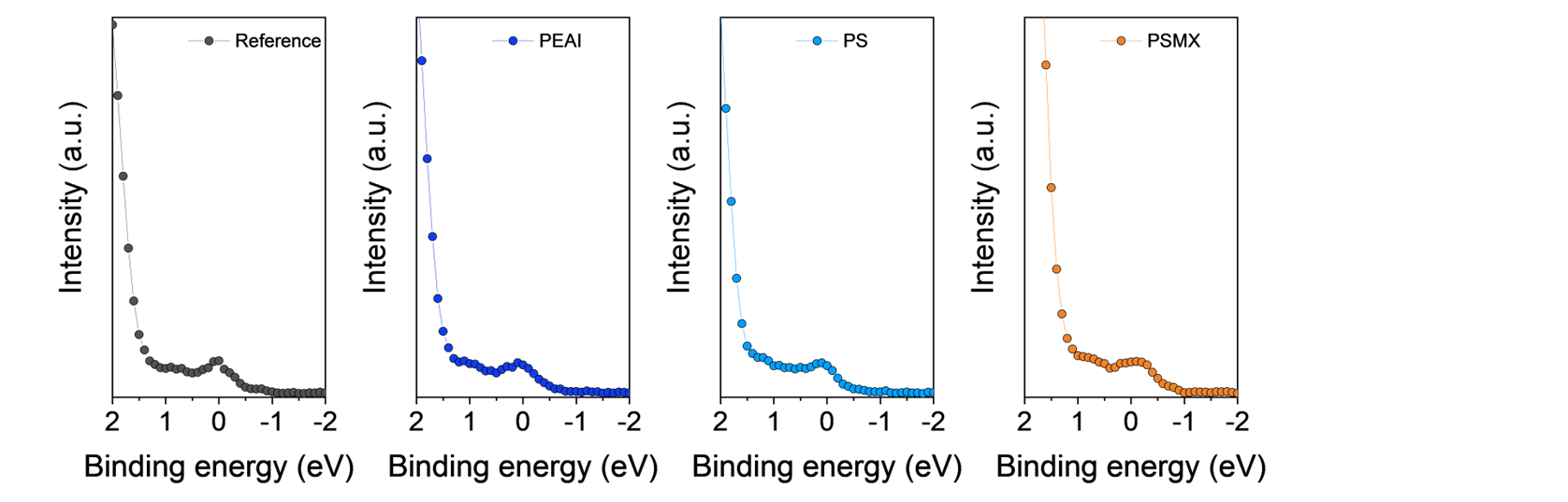


**Figure S2.** UPS spectra of perovskite films with different surface treatment at lower energy.


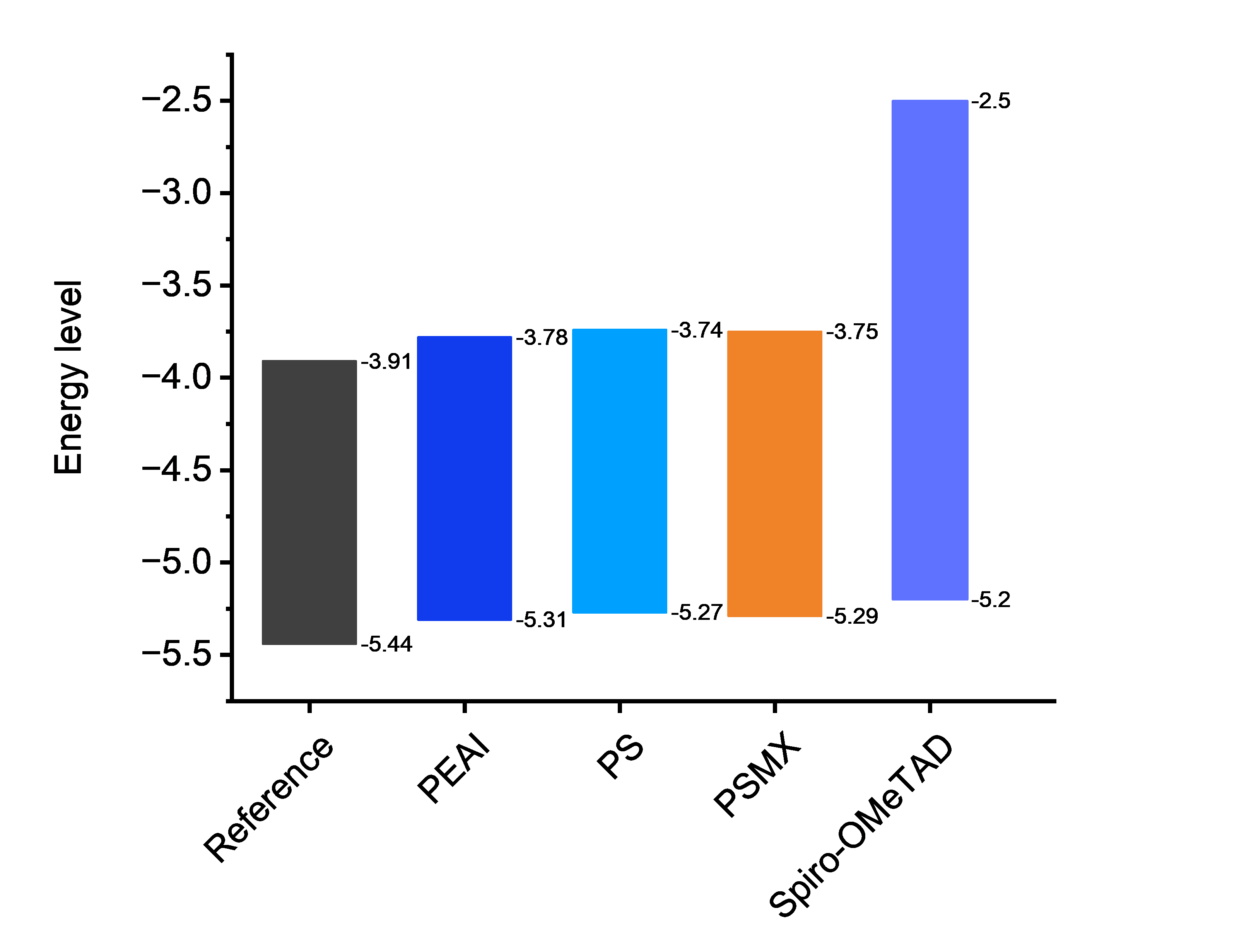


**Figure S3**. Energy level diagram of perovskites treated with different materials.


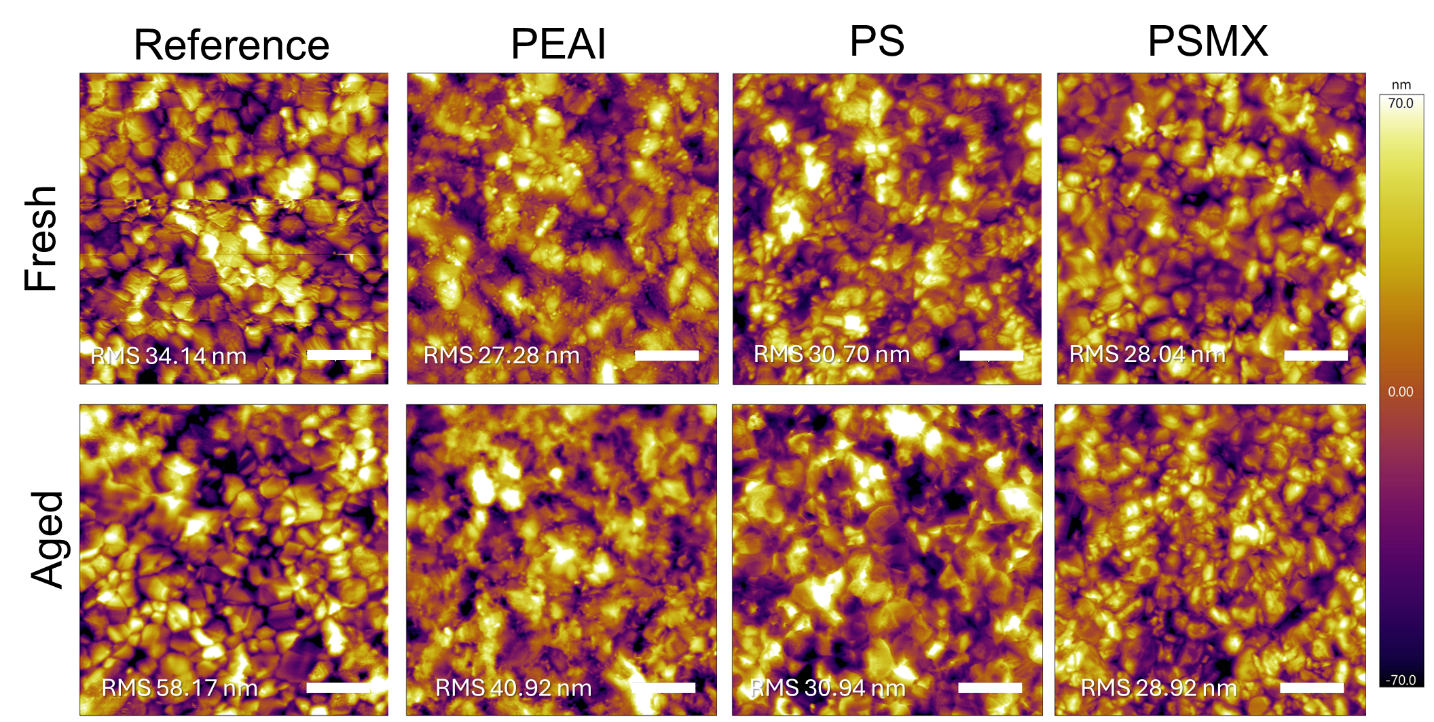


**Figure S4.** AFM images of perovskite films with various surface passivation treatments, conducted before and after aging under ambient conditions (RH ~75%) for 2 hours (scale bar: 2 µm).


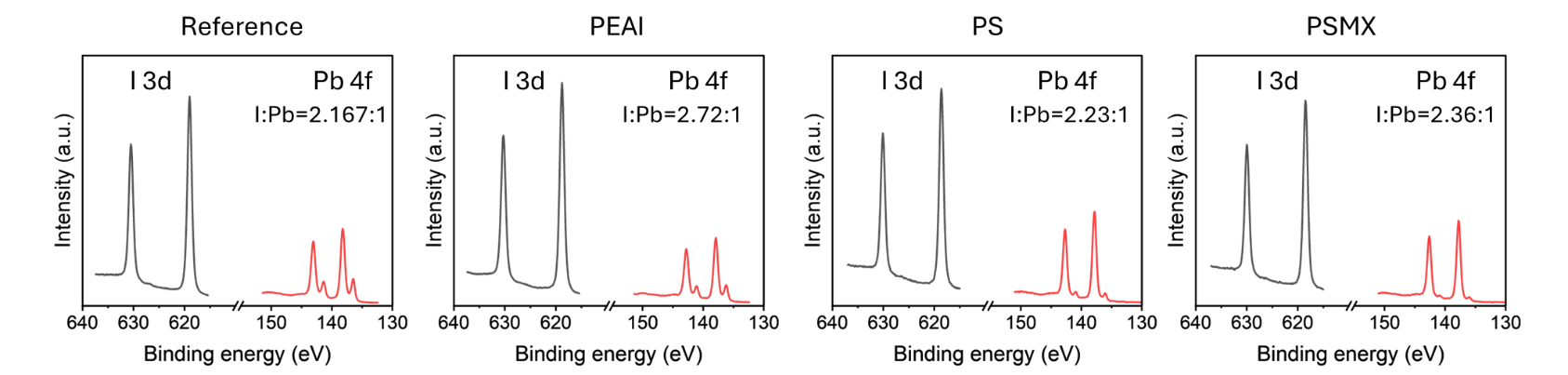


**Figure S5.** HR XPS Pb 4f and I 3d core energy level spectra of α-FAPbI_3_ without a) (reference) and with b) PEAI, c) PS and d) PSMX passivation.


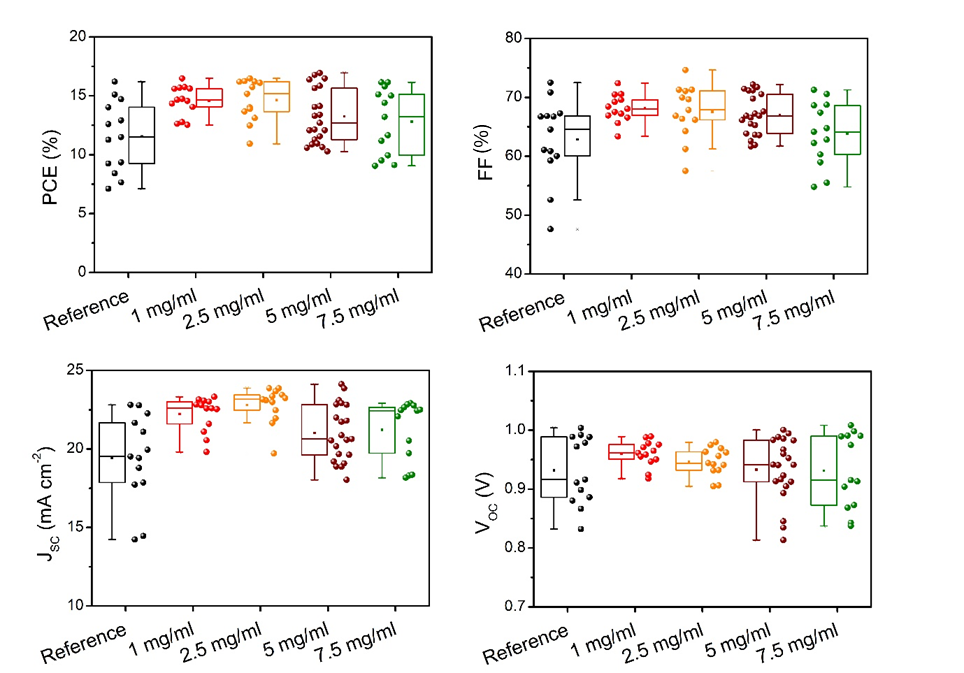


**Figure S6.** Statistical distribution of PCE, FF, J_SC_ and V_OC_ of PSCs without (reference) and with PS at different concentrations ranging from 1 mg mL^-1^ to 7.5 mg mL^-1^.


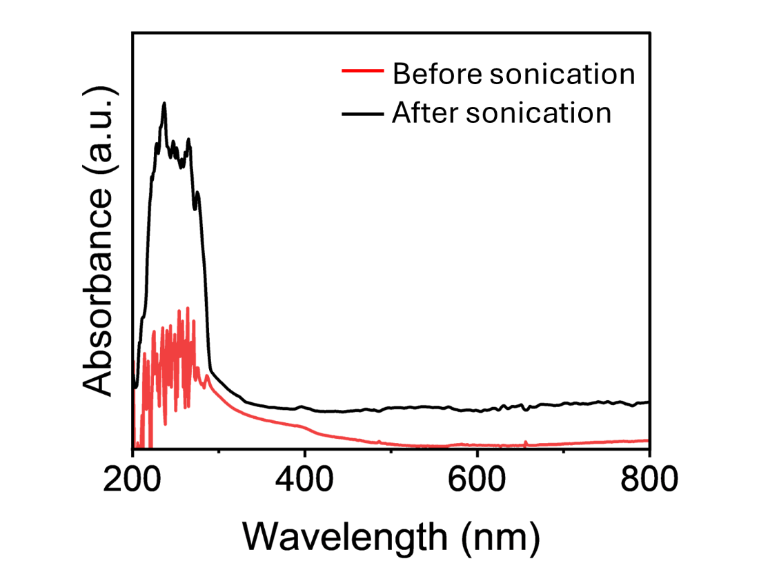


**Figure S7.** UV-vis spectra of Ti_3_C_2_T_x_ solution in chlorobenzene with a concentration of 0.5 mg/mL before and after 30 min sonication.


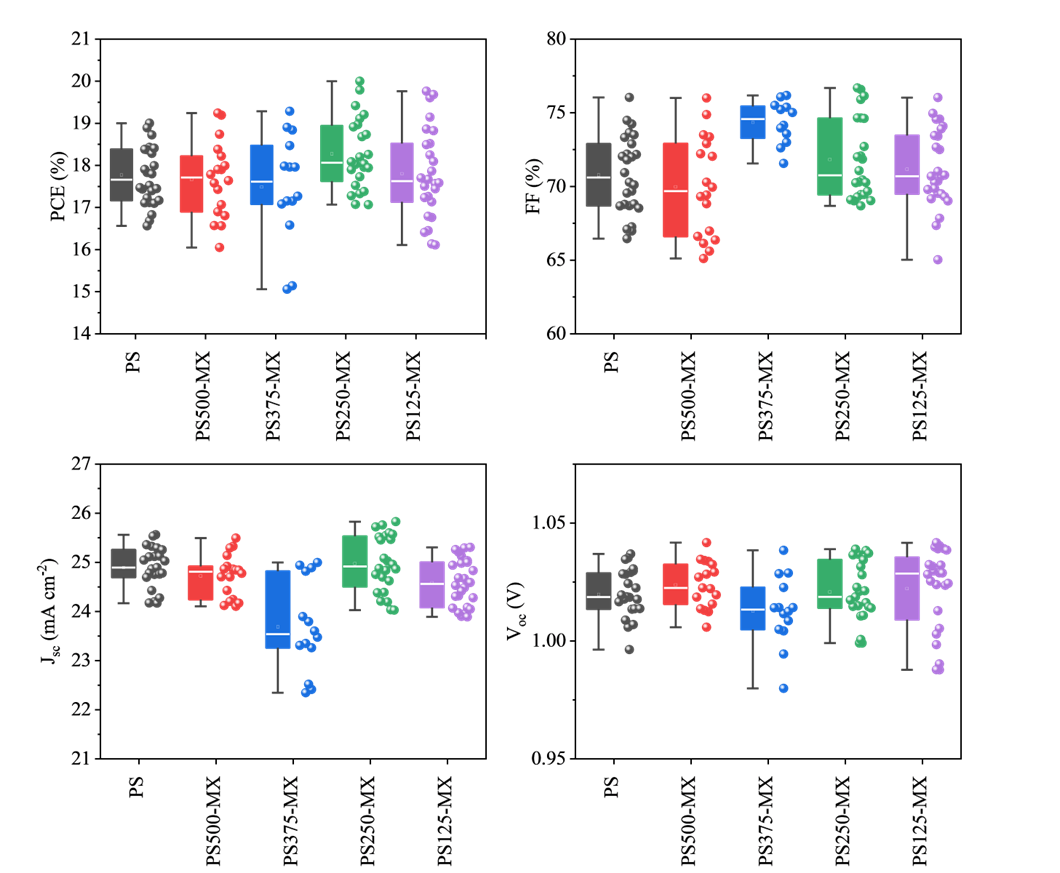


**Figure S8.** Statistical distribution of PCE, FF, J_SC_ and V_OC_ of PSCs with PSMX passivation at different mass ratio of PS:MX ranging from 500:1, 375:1, 250:1 and 125:1.


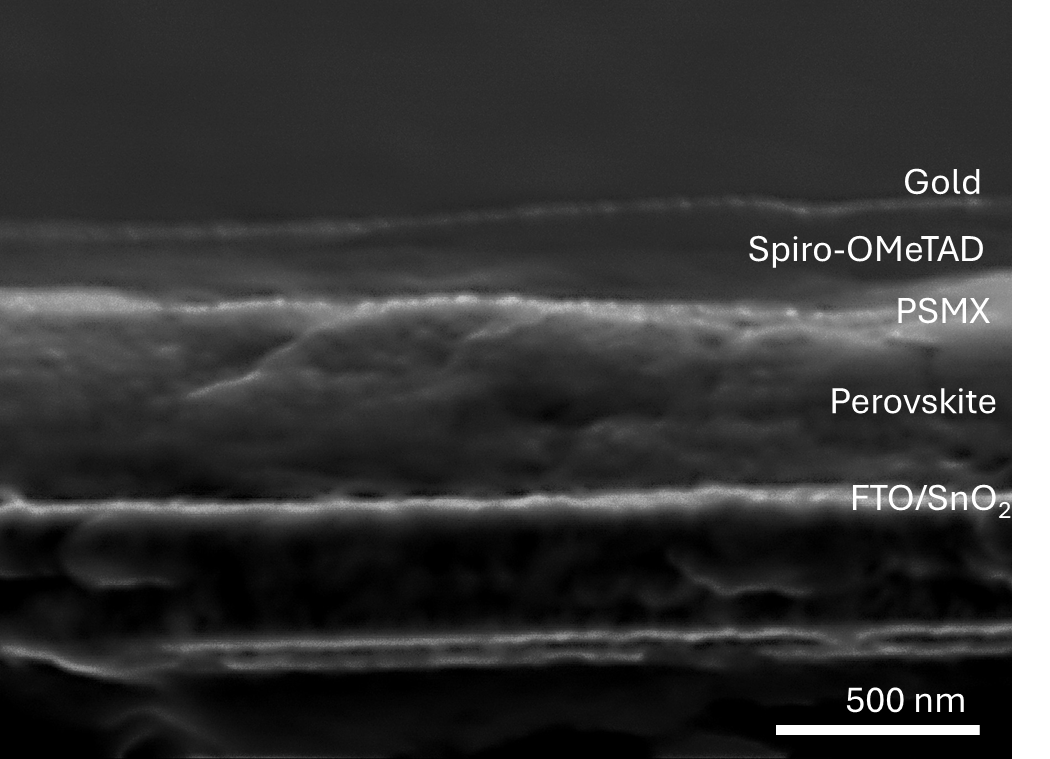


**Figure S9.** Cross sectional SEM image of the fabricated PSC with PSMX passivation (250:1).


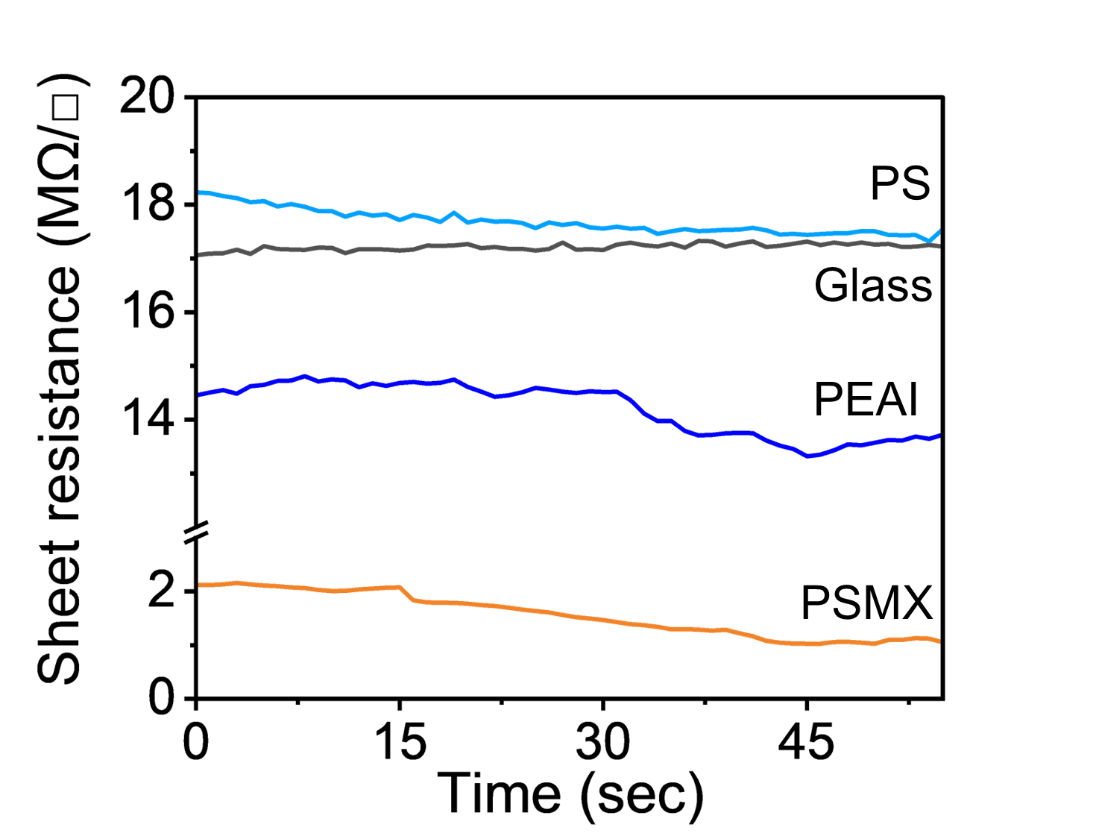


**Figure S10.** Sheet resistance of bare glass and PEAI, PS and PSMX coated glass substrates.


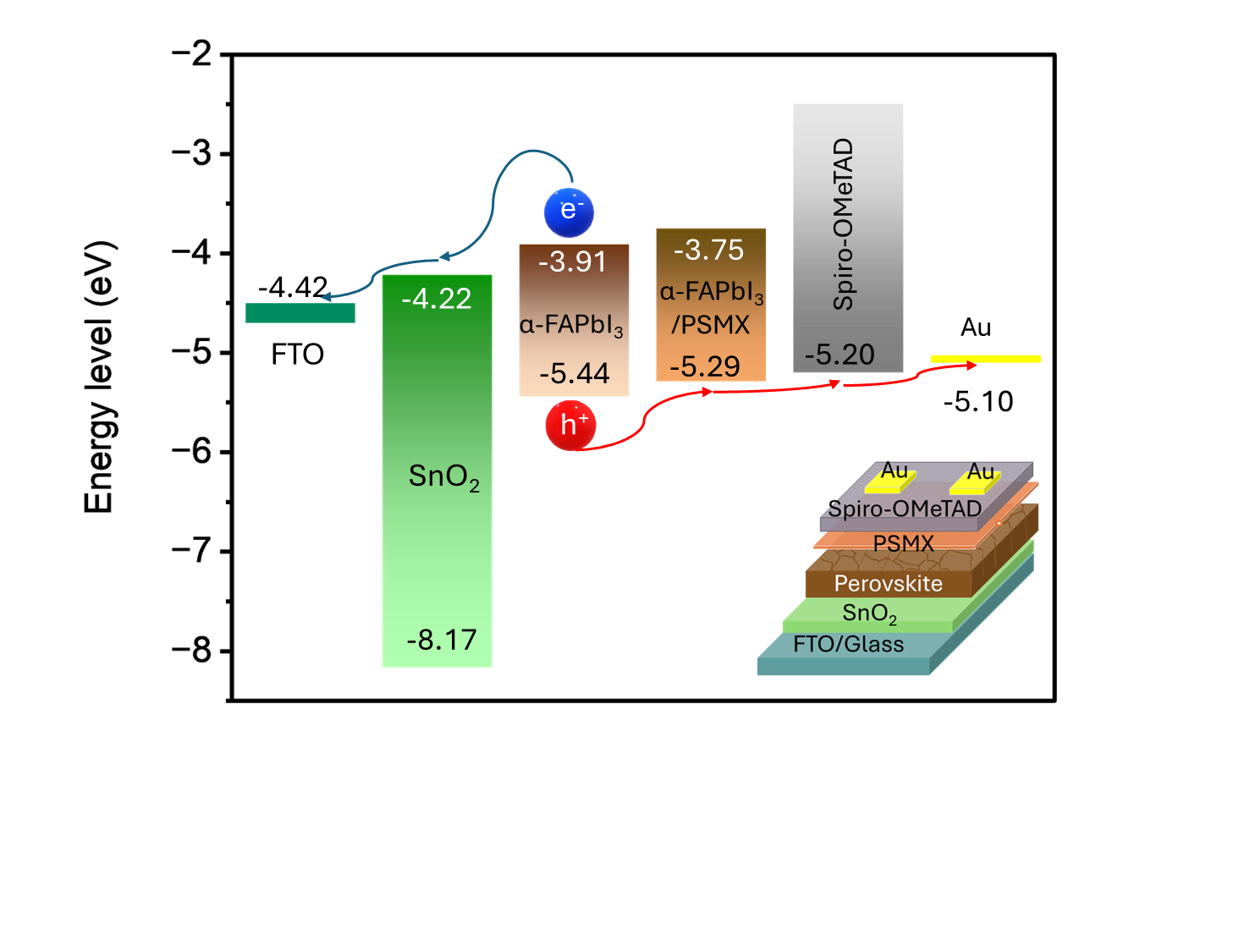


**Figure S11.** Energy level diagram of a full PSC device with PSMX passivation.


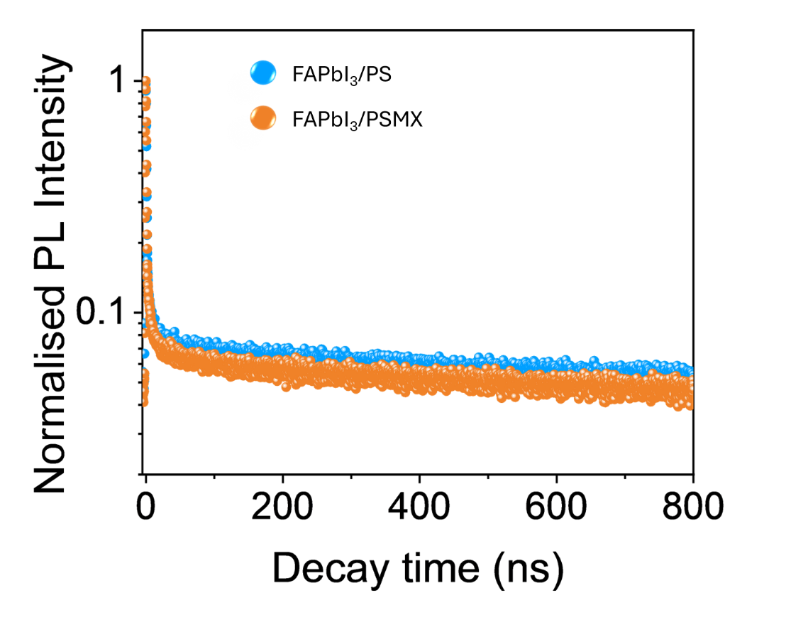


**Figure S12.** TRPL decays of perovskite films passivated with PS (blue) and PSMX (orange) on glass substrate.


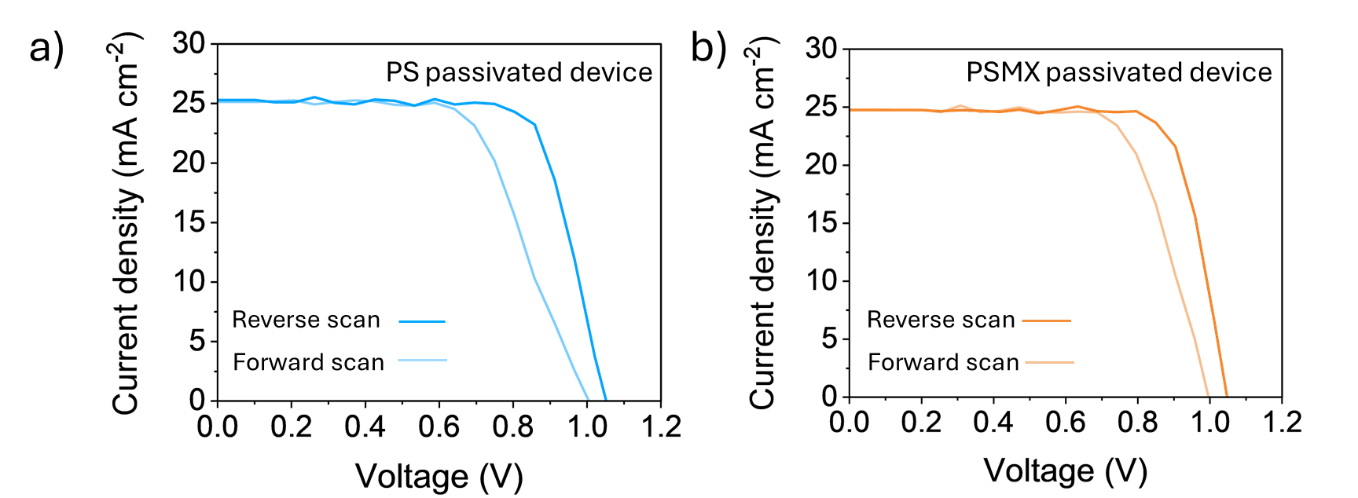


**Figure S13.** J–V characteristics of the a) PS and b) PSMX passivated devices measured in the reverse and forward scan directions at 150 mV s^-1^.


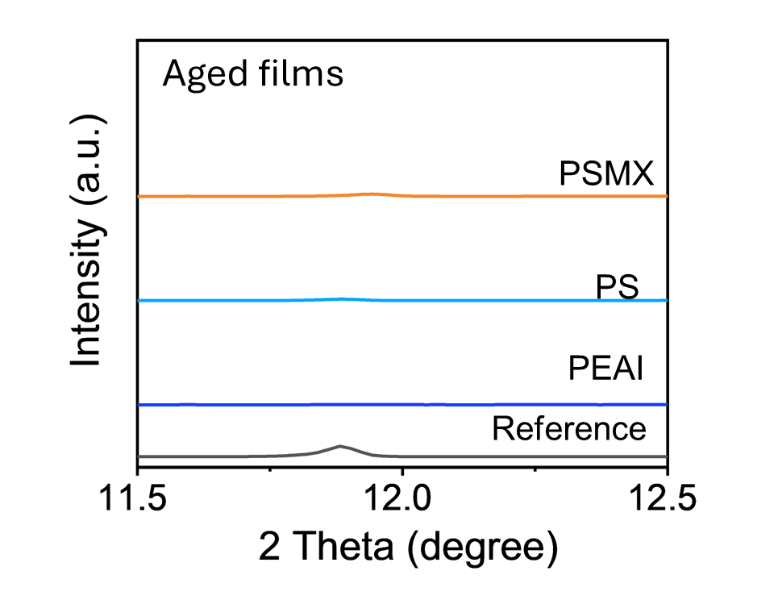


**Figure S14.** XRD patterns of aged films in narrow range of 11.5°- 12.5° at ~75% RH for 2 h.


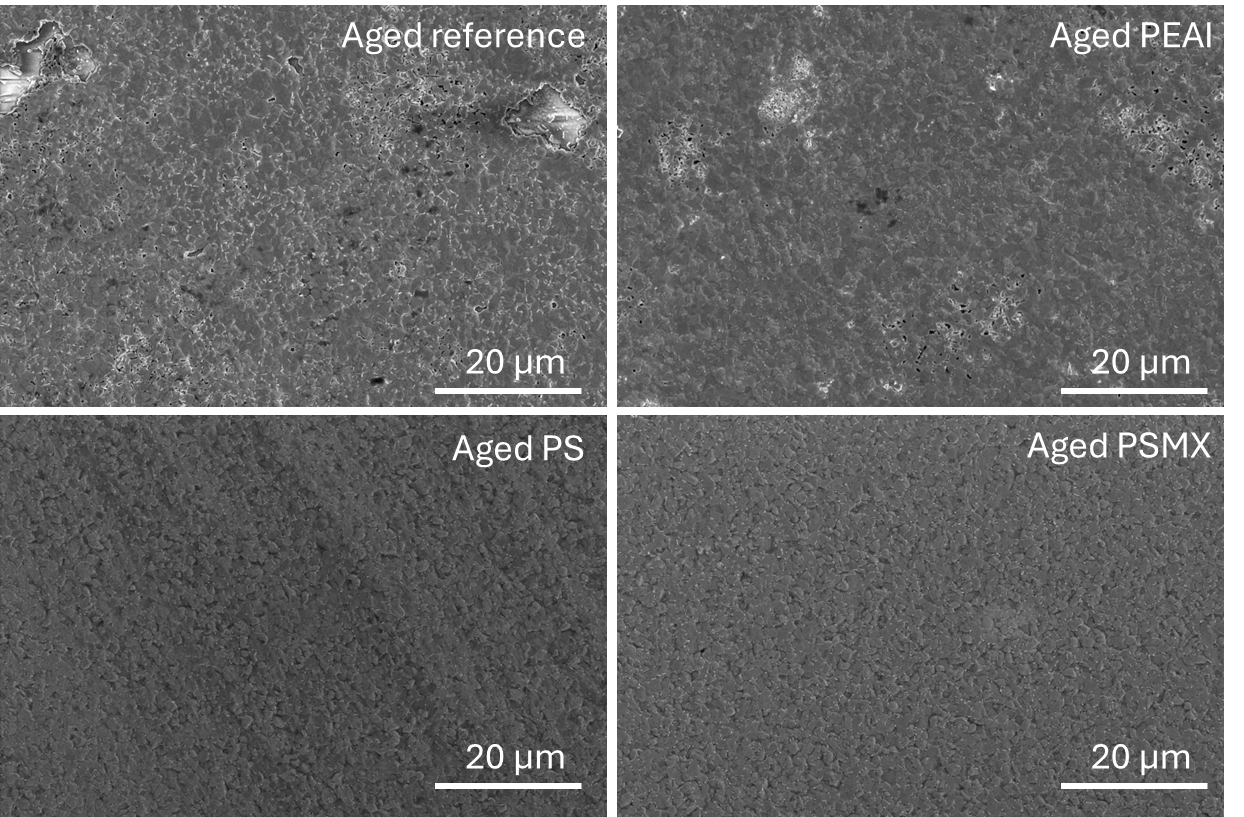


**Figure S15.** Top-view SEM images of aged reference, PEAI, PS and PSMX passivated perovskite films.

**References**

[1] R. J. Jin, Y. H. Lou, L. Huang, K. L. Wang, C. H. Chen, J. Chen, F. Hu and Z. K Wang, Photochemical Shield Enabling Highly Efficient Perovskite Photovoltaics, *Advanced Materials*, **2024**, *36*, 2313154.

[2] C. J. Zhang, S. Pinilla, N. McEvoy, C. P. Cullen, B. Anasori, E. Long, S.-H. Park, A. Seral-Ascaso, A. Shmeliov, D. Krishnan, C. Morant, X. Liu, G. S. Duesberg, Y. Gogotsi and V. Nicolosi, *Oxidation Stability of Colloidal Two-Dimensional Titanium Carbides (MXenes),* *Chemistry of Materials*, **2017**, *29*, 4848-4856.
